# International Physical and Rehabilitation Medicine Training in France: Experiences of African Physicians

**DOI:** 10.7759/cureus.110682

**Published:** 2026-06-11

**Authors:** Tendart D Viannel, Mounguengui Hartwig, Ammari Maryeme

**Affiliations:** 1 Department of Physical Medicine and Rehabilitation, Hopital Militaire d'Instruction Mohammed V, Rabat, MAR

**Keywords:** africa, international training, medical eduation, physical medicine and rehabilitation, rehabilitation workforce development

## Abstract

Objective

To explore the self-reported clinical, educational, technical, and scientific experiences of African physicians undertaking Physical and Rehabilitation Medicine (PRM) training in France.

Design

This study was designed as a descriptive cross-sectional multicenter study.

Subjects

The subjects included African physicians who completed a PRM clinical training placement in France between 2016 and 2024.

Methods

An anonymous online questionnaire was distributed between February and April 2025. Collected data included clinical activities, exposure to interventional procedures, participation in educational and scientific activities, overall satisfaction, and perceived areas for improvement. Data were analyzed descriptively.

Results

Twenty-five physicians responded to the survey. Clinical exposure was heterogeneous and predominantly focused on neurorehabilitation. Limited exposure was reported in spinal cord injury rehabilitation, complex prosthetics and orthotics, and amputee rehabilitation. Exposure to interventional procedures varied across training sites. Botulinum toxin injections and corticosteroid injections were the most frequently reported procedures, whereas more specialized interventions remained uncommon. Participation in educational activities was generally satisfactory, while involvement in research activities remained limited. Overall satisfaction with the training experience was high.

Conclusion

PRM training placements in France provide a valuable educational experience for African physicians. However, significant heterogeneity in clinical and technical exposure highlights the need for improved educational structuring and harmonization of training pathways. These findings support the establishment of minimum practical and educational objectives for international PRM training programs.

## Introduction

Physical and Rehabilitation Medicine (PRM) is a medical specialty focused on improving functioning and participation in people living with disability or functional limitations related to health conditions [1].

Africa currently faces a shortage of rehabilitation professionals, especially PRM physicians, despite increasing rehabilitation needs and the growing burden of disabling conditions [2-4]. Developing rehabilitation services therefore requires better training opportunities for healthcare professionals. In this context, France represents an important training destination for physicians from Francophone African countries seeking PRM training.

These training opportunities are mainly organized through three pathways: associated clinical placements (“stage associé”), the Diplôme de Formation Médicale Spécialisée (DFMS), and the Diplôme de Formation Médicale Spécialisée Approfondie (DFMSA). These programs differ in duration and academic organization. However, limited data are available describing the actual clinical, educational, technical, and scientific experiences of African physicians undertaking PRM training in France.

The aim of this study was to explore the experiences of African physicians who completed PRM training placements in France between 2016 and 2024, focusing on self-reported clinical exposure, interventional procedures, educational activities, scientific involvement, overall satisfaction, and perceived areas for improvement.

## Materials and methods

Study design

This was a descriptive, cross-sectional, multicenter study.

Study population

African physicians who completed a PRM clinical training placement in France between 2016 and 2024, lasting at least one semester, were eligible for inclusion.

Eligible participants were identified through professional networks and the African PRM physicians’ association. The survey was distributed by email and WhatsApp, with reminder messages sent during the study period. The questionnaire was pre-tested before dissemination.

Data collection

Data were collected using an anonymous online questionnaire created with Google Forms and distributed between February and April 2025.

The questionnaire used in this study was developed by the authors and is provided in Appendix 1.

The questionnaire collected data on demographic characteristics, type and duration of training, clinical exposure across PRM domains, participation in educational activities, exposure to interventional procedures, involvement in scientific activities, overall satisfaction, and perceived areas for improvement.

All data were self-reported. The objective of the study was not to assess competency acquisition objectively, but rather to describe the participants’ perceived educational and clinical experiences during their training in France.

Outcome measures

The main outcomes assessed were clinical exposure across PRM domains, exposure to interventional procedures, access to educational and scientific activities, overall satisfaction, and perceived areas for improvement.

Statistical analysis

Data were analyzed descriptively using Microsoft Excel. Categorical variables were expressed as frequencies and percentages.

No inferential statistical analyses were performed because the study was descriptive in nature.

## Results

Response rate

The questionnaire was distributed to 35 trainees, and 25 responses were collected, corresponding to a response rate of 71.4%.

Participants’ characteristics

**Figure 1:**
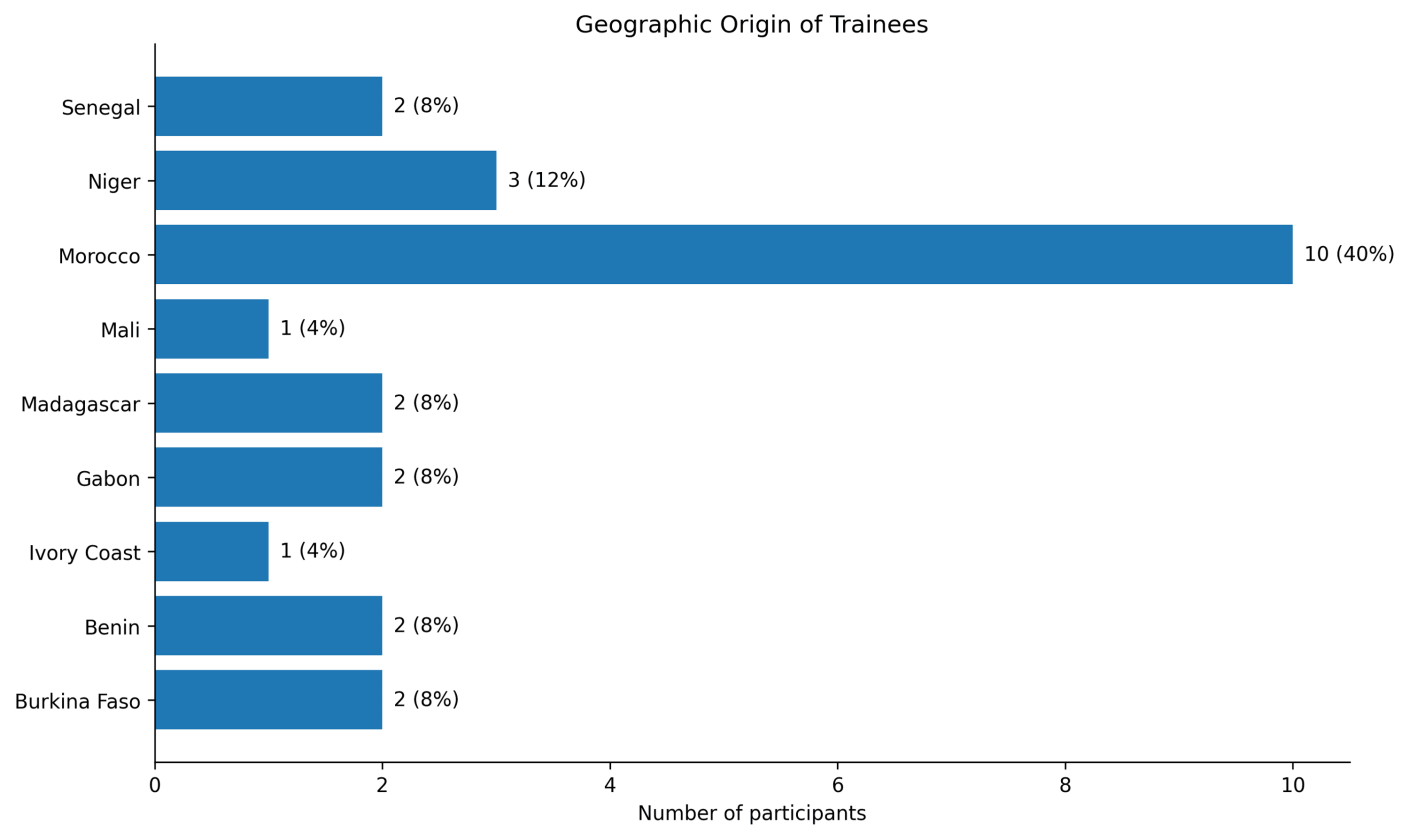
Geographic origin of trainees. Data are presented as the number of participants (n) and percentages (%).

Twenty-five African physicians responded to the questionnaire. Most participants originated from North Africa and West Africa (Figure 1).

Training characteristics

**Figure 2:**
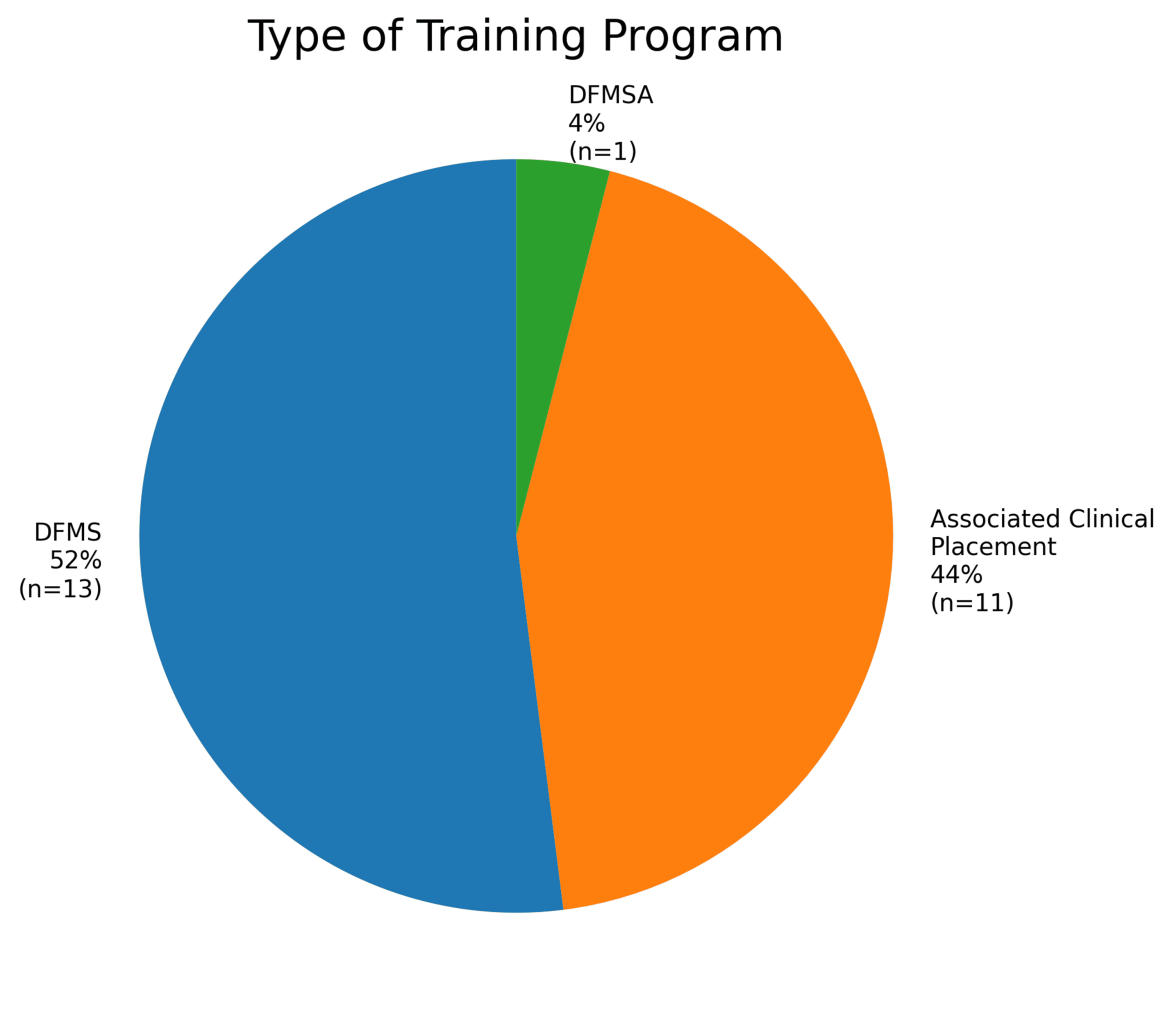
Type of training program. Data are presented as the number of participants (n) and percentages (%).

More than half of the respondents had completed their training under a DFMS program (Figure 2). Training duration and host institutions varied across participants.

Clinical and educational activities

Clinical exposure across PRM domains is presented in Table 1. Clinical exposure was heterogeneous, with a predominance of neurological rehabilitation: 80% of trainees reported neurological rehabilitation activities accounting for more than 50% of their clinical time, including 12% who reported more than 75%. Orthopaedic and musculoskeletal rehabilitation was less prominent, with 60% reporting exposure above 50%, including 16% reporting exposure above 75%.

**Table 1:** Percentage of clinical exposure according to Physical and Rehabilitation Medicine domains. Data are presented as percentages (%) of trainees (n = 25).

Domain	<50% (%)	50-75% (%)	>75% (%)
Neurological rehabilitation	20	68	12
Orthopaedic rehabilitation	40	44	16
Spinal cord injury rehabilitation	96	4	0
Complex prosthetics and orthotics	72	16	12
Amputee rehabilitation	68	28	4

In contrast, some specific PRM fields were poorly accessible. Spinal cord injury rehabilitation accounted for less than 50% of activity in 96% of participants, as did complex prosthetics and orthotics consultations for 72%. Amputee rehabilitation was also limited, with 68% of trainees reporting less than 50% exposure and only 4% reporting more than 75%.

Interventional procedures

Regarding interventional procedures, results showed heterogeneous practice depending on the training site. Botulinum toxin injections were the most frequently performed procedure, with participation reported by 60% of trainees (n = 15), and 44% of all trainees (n = 11) reported performing six or more injections. Corticosteroid injections were also relatively common, with 40% of trainees (n = 10) reporting at least one infiltration, including 8% (n = 2) who performed more than 10 procedures.

Conversely, specialised procedures remained poorly accessible. Arthrodistension was reported by only 20% of trainees (n = 5), mostly with limited exposure. Nerve blocks were also reported by 20% (n = 5). Finally, intrathecal baclofen pump management remained marginal, reported by 24% of trainees (n = 6) (Table 2).

**Table 2:** Exposure to interventional PRM procedures during training. Data are presented as the number of trainees (n) and percentages (%). PRM: Physical and Rehabilitation Medicine.

Procedure	0 procedures, n (%)	1-5 procedures, n (%)	6-10 procedures, n (%)	>10 procedures, n (%)
Corticosteroid injections	15 (60%)	8 (32%)	0 (0%)	2 (8%)
Botulinum toxin injections	10 (40%)	4 (16%)	2 (8%)	9 (36%)
Arthrodistension	20 (80%)	4 (16%)	1 (4%)	0 (0%)
Nerve blocks	20 (80%)	4 (16%)	1 (4%)	0 (0%)
Intrathecal baclofen pump management	19 (76%)	6 (24%)	0 (0%)	0 (0%)

Educational, academic, and scientific activities 

Educational and Academic Activities

A majority of respondents reported having benefited from educational activities during their training, with 64% (n = 16) attending theoretical courses or formal teaching sessions. In addition, 48% of trainees (n = 12) participated in at least one COFEMER training module.

Teaching topics covered a wide spectrum of PRM, including neurological and musculoskeletal rehabilitation, spinal cord injury management, orthotics and prosthetics, amputations, paediatric PRM, chronic pain, pressure ulcers, gait and balance disorders, and functional assessment in PRM.

Integration into multidisciplinary clinical activities was frequent, with 72% of trainees (n = 18) participating in multidisciplinary consultations or case discussions. However, participation in journal clubs was less frequent, reported by only 36% (n = 9).

Research and Scientific Activities

Participation in research activities or scientific production was reported by 20% of trainees (n = 5). These activities mainly consisted of scientific communications, such as posters or oral presentations at conferences, or participation in manuscript writing.

Overall satisfaction and recommendations

Most trainees reported a globally positive training experience, with a high level of satisfaction. More than two-thirds of respondents rated their overall satisfaction between 4 and 5 out of 5. Only two trainees reported a satisfaction score of 2 or less.

The most frequently reported positive aspects included quality of supervision, availability of medical and paramedical teams, work atmosphere, and team spirit. Technical facilities, diversity of clinical cases, and progressive clinical responsibility were also commonly highlighted.

Conversely, the main areas for improvement concerned the pedagogical structuring of the training. Many trainees expressed the need for harmonisation of DFMS training, as well as reinforcement of theoretical and practical teaching. Lack of structured training in interventional procedures was one of the most frequently reported issues. Other expectations included better formalised initial orientation, improved access to research activities, and better recognition of foreign physicians within clinical teams.

## Discussion

This study explored the experiences of African physicians who completed PRM training placements in France between 2016 and 2024. Overall, participants reported a positive educational experience, particularly regarding supervision, integration within clinical teams, and exposure to neurorehabilitation. However, the results also showed important heterogeneity in clinical and technical exposure depending on the training site.

These findings should be interpreted in the broader context of increasing rehabilitation needs worldwide, especially in low- and middle-income countries, where access to rehabilitation professionals remains limited. International training opportunities may therefore contribute to strengthening the rehabilitation workforce in African countries [5].

To our knowledge, this study is among the first to specifically describe the clinical, educational, technical, and scientific experiences of African physicians undertaking PRM training in France. Most available publications focus on rehabilitation workforce shortages or PRM education in general, rather than on the experiences of international trainees [3,6-8].

Clinical exposure and interventional procedures

In our study, neurorehabilitation represented the main area of clinical exposure. This finding is consistent with the central role of neurological rehabilitation in many PRM departments and training centers [1,9]. In contrast, exposure to spinal cord injury rehabilitation, amputee rehabilitation, and complex prosthetics and orthotics remained limited for many trainees. Similar disparities in access to specific PRM fields have been described in other international training settings [5].

Exposure to interventional procedures also varied considerably between training sites. Botulinum toxin injections and corticosteroid injections were the most accessible procedures, whereas arthrodistension, nerve blocks, and intrathecal baclofen pump management remained uncommon. This variability probably reflects differences in local organization, available equipment, and supervisor expertise. The absence of clearly defined practical objectives for trainees may also contribute to these differences. Similar heterogeneity has been reported in PRM training programs in other regions [10,11].

Educational and scientific activities

Most participants reported satisfactory access to educational activities, including theoretical teaching and multidisciplinary discussions. However, participation in research activities remained limited. This finding has also been reported in studies involving international medical trainees and may reflect limited academic integration, language barriers, or a lack of dedicated research supervision [9,10].

Overall satisfaction and perspectives for improvement

Despite these limitations, overall satisfaction was high. Participants mainly highlighted the quality of supervision, team atmosphere, diversity of clinical cases, and progressive clinical responsibility as positive aspects of their training experience. At the same time, many trainees expressed the need for better harmonization of training pathways, more structured practical teaching, and clearer educational objectives.

These findings suggest that international PRM training programs may benefit from a more standardized educational framework, including minimum clinical and technical competencies to be achieved during training. Such measures could help reduce disparities between training sites and improve long-term skill transfer to countries with limited rehabilitation resources.

Limitations

This study has several limitations. First, the sample size was relatively small. Second, the study relied on self-reported data, reflecting participants’ perceptions and experiences rather than objective measures of competency acquisition, and may have introduced recall bias. In addition, participants trained in different institutions with heterogeneous clinical practices and educational organization. The questionnaire used in this study was not formally validated. Finally, the possibility of selection bias and non-response bias cannot be excluded.

Despite these limitations, the study provides original data on a poorly described population and highlights several important aspects of international PRM training experiences among African physicians.

## Conclusions

African physicians undertaking PRM training placements in France generally reported a positive clinical and educational experience. Strengths of these programs included the quality of supervision, exposure to neurorehabilitation, multidisciplinary practice, and progressive clinical responsibility.

However, important disparities were identified regarding access to certain PRM subspecialties, interventional procedures, and research activities. These findings highlight the need for better harmonization of training pathways and the establishment of minimum educational and practical objectives for international PRM training programs.

Improving the organization and educational structure of these training experiences could contribute to strengthening rehabilitation medicine development and workforce capacity in African countries.

## Appendices

**Table 3:** English translation of the survey questionnaire. PRM: Physical and Rehabilitation Medicine; DFMS: Diplôme de Formation Médicale Spécialisée; DFMSA: Diplôme de Formation Médicale Spécialisée Approfondie; CHU: University hospital; ENMG: Electroneuromyography; PRP: Platelet-rich plasma.

Item	Survey question	Response options
1	What is your nationality?	Open-ended
2	What is your home university for PRM training?	Open-ended
3	In which year did you come to France for your training placement?	Open-ended
4	How many PRM semesters had you completed before coming to France?	1-2; 3-4; 5-6; 7-8; >8
5	Under which framework was your placement conducted?	Associated clinical placement; DFMS; DFMSA
6	In which city/cities did you complete your placement?	Open-ended
7	In which institution(s) did you complete your placement?	Open-ended
8	What type of institution hosted your placement?	University hospital (CHU); general hospital (CH); private hospital; other
9	During your placement, what proportion of your clinical activity involved neurological rehabilitation?	<50%; 50-75%; >75%
10	During your placement, what proportion of your clinical activity involved orthopaedic rehabilitation?	<50%; 50-75%; >75%
11	During your placement, what proportion of your clinical activity involved spinal cord injury rehabilitation?	<50%; 50-75%; >75%
12	During your placement, what proportion of your activity involved consultation for and prescription of major orthotic and prosthetic devices?	<50%; 50-75%; >75%
13	During your placement, how many corticosteroid, PRP, hyaluronic acid, or dextrose injections did you perform?	0; 1-5; 6-10; >10
14	During your placement, how many botulinum toxin injection procedures did you perform?	0; 1-5; 6-10; >10
15	During your placement, how many shoulder hydrodilatation procedures or knee viscosupplementation procedures did you perform?	0; 1-5; 6-10; >10
16	During your placement, how many nerve block procedures did you perform?	0; 1-5; 6-10; >10
17	Which equipment, techniques, or procedures were you exposed to or trained in?	Musculoskeletal ultrasound; isokinetic assessment; electroneuromyography (ENMG); urodynamic assessment; functional electrical stimulation; gait analysis platform; posturography platform; driving simulator; other
18	Did you receive formal teaching sessions during your placement?	Yes; No
19	If yes, please provide examples of topics covered during these teaching sessions.	Open-ended
20	Did you attend COFEMER educational modules?	Yes; No
21	If yes, please specify the modules attended.	Open-ended
22	Did you participate in multidisciplinary consultations or multidisciplinary team meetings?	Yes; No
23	Did you participate in research activities or scientific publications during your placement?	Yes; No
24	If yes, please specify the topic and type of scientific activity, such as an article, poster, or oral presentation.	Open-ended
25	Did you participate in journal clubs or bibliographic review meetings?	Yes; No
26	How would you rate the infrastructure and equipment available at your training site?	Likert scale (1-5)
27	How would you rate the organization of the healthcare team, including communication and collaboration?	Likert scale (1-5)
28	How would you rate the quality of supervision during your placement?	Likert scale (1-5)
29	Please describe the positive aspects of your placement experience.	Open-ended
30	Please describe aspects of the placement that could be improved.	Open-ended
31	How likely are you to recommend this training experience to other trainees?	Likert scale (1-5)

## References

[1] European Physical and Rehabilitation Medicine Bodies Alliance (2018). White Book on Physical and Rehabilitation Medicine (PRM) in Europe. Chapter 1. Definitions and concepts of PRM. Eur J Phys Rehabil Med.

[2] Mapulanga M, Dlungwane T (2022). Mapping evidence of community health workers delivering physical rehabilitation services in sub-Saharan Africa: a scoping review protocol. BMJ Open.

[3] Tannor AY, Nelson ME, Steere HK, Quao BO, Haig AJ (2022). Building PRM in sub-Saharan Africa. Front Rehabil Sci.

[4] Mouguina I (2022). Médecine physique et rééducation fonctionnelle (MPR) au Maroc et en Afrique: état des lieux et de connaissances [doctoral thesis]. Marrakech: Faculté de médecine et de pharmacie, Université Cadi Ayyad.

[5] Ceravolo MG, Gimigliano F, Lains J (2023). Editorial: pursuing quality education in physical and rehabilitation medicine. Front Rehabil Sci.

[6] World Health Organization (2017). [6] World Health Organization. Rehabilitation 2030: a call for action. Rehabilitation 2030: a call for action.

[7] Cieza A, Causey K, Kamenov K, Hanson SW, Chatterji S, Vos T (2021). Global estimates of the need for rehabilitation based on the Global Burden of Disease study 2019: a systematic analysis for the Global Burden of Disease Study 2019. Lancet.

[8] Mathews A, Doobay-Persaud A, Rydberg L (2021). The development of a novel international elective in Physical Medicine and Rehabilitation. Am J Phys Med Rehabil.

[9] European Physical and Rehabilitation Medicine Bodies Alliance (2018). White Book on Physical and Rehabilitation Medicine (PRM) in Europe. Chapter 9. Education and continuous professional development: shaping the future of PRM. Eur J Phys Rehabil Med.

[10] Petriello M, Mathews A, Eison K, Hartman E, Steere H (2023). Survey of global health education in Physical Medicine and Rehabilitation residency programs in the United States. J Int Soc Phys Rehabil Med.

[11] Jin Y, Ma L, Zhou J, Xiong B, Fernando A, Snelgrove H (2024). A call for improving of musculoskeletal education on physical medicine and rehabilitation studies: a systematic review with meta-analysis. BMC Med Educ.

